# Development of SFC-MS Method for Quantification of Eicosanoids Biosynthesized in Primary Human Blood Cells

**DOI:** 10.3390/metabo12121198

**Published:** 2022-11-30

**Authors:** Louis Schmidt, Laura Sophie Burmeister, Andreas Greinacher, Stefanie König, Ulrike Garscha

**Affiliations:** 1 Department of Pharmaceutical/Medicinal Chemistry, Institute of Pharmacy, Greifswald University, 17489 Greifswald, Germany; 2 Institute of Transfusion Medicine, University Medicine Greifswald, 17489 Greifswald, Germany

**Keywords:** oxylipins, supercritical fluid chromatography, monocytes, neutrophils, platelets, validation, lipid mediators

## Abstract

Eicosanoids are lipid mediators generated from arachidonic acid with pro- and anti-inflammatory properties. Despite these lipid mediators being known for decades, quantitative determination in biological samples is still challenging due to low abundance, instability, the existence of regio- and stereoisomers, and a wide polarity range that hampers chromatographic separation. In this study, we developed a supercritical fluid chromatography mass spectrometry (SFC-MS) platform for the quantification of relevant eicosanoids. Application of a chiral amylose-based column and modifier combination of 2-propanol/acetonitrile offered separation and sufficient resolution of 11 eicosanoids (5-, 12-, 15-HETE, PGB_1_, LTB_4_, t-LTB_4_, 20-OH-LTB_4_, PGE_2_, PGD_2_, PGF_2α_, TxB_2_) with baseline separation of isobaric analytes within 12 min. The method was validated in terms of range (78–2500 ng/mL), linearity, accuracy, precision, and recovery according to EMA guidelines. Finally, we confirmed the method’s applicability by quantifying eicosanoid levels in human primary blood cells. In conclusion, we present a validated SFC-MS method for the determination of relevant eicosanoids in biological samples with a wide range of polarity while maintaining baseline separation of isobars, which allows coupling to a single quadrupole mass detector.

## 1. Introduction

Eicosanoids, including prostaglandins (PGs), leukotrienes (LTs), and thromboxanes (TXs) comprise a group of oxylipins biosynthesized from arachidonic acid (AA) and are involved in several physiological but also pathophysiological processes [1]. Upon release from phospholipids by cytosolic phospholipase A2 (cPLA_2_), AA is dioxygenated by cyclooxygenases 1/2 (COX-1/2) [2] and lipoxygenases (LOXs) [3] or monooxygenated by cytochrome (CYP) P450 enzymes [4,5]. PGs such as PGE_2_, PGF_2α_, PGD_2_, and TXs are formed by tissue- and cell-specific downstream synthases from PGH_2_, which itself is biosynthesized by COX-1/2. PGE_2_ is one of the most abundant PGs with an impact on fertility and gastric mucosal integrity but also on inflammation, fever, and cancer development [6,7]. While PGD_2_ is synthesized in the central nervous system regulating sleep and other CNS activities [8] and also in peripheral tissues, especially in mast cells and other leukocytes, PGF_2α_ regulates ovulation and contraction of uterine smooth muscle and initiates parturition [7]. Furthermore, in activated platelets, significant amounts of TXs are produced from PGH_2_ acting in an autocrine and paracrine manner with potent prothrombotic and vasoconstrictive properties [9]. In addition to COX-derived PGs and TXs, LTs are biosynthesized via the 5-LOX pathway [10]. 5-LOX dioxygenates AA to 5-hydroperoxyeicosatetraenoic acid (5-HPETE) and in a subsequent step to the unstable epoxide LTA_4_. This intermediate is enzymatically further metabolized to LTB_4_ and LTC_4_ by LTA_4_-hydrolase (LTA_4_H) and LTC_4_-synthase (LTC_4_S) [3], respectively. While LTB_4_ belongs to the most chemotactic compounds and is predominately formed in neutrophils, LTC_4_ is known as a slow-reacting substance of anaphylaxis leading to bronchoconstriction during allergy and asthma [3]. Demonstrating the versatile importance of eicosanoids, which was discovered in the last four decades, rapid and validated methods are required to detect and quantify those oxylipins in complex biological samples to enhance the understanding of the lipid mediator network during physiological and pathophysiological conditions. However, the often-neglected fact of autoxidation, isomer formation, and instability complicate the detection and hamper the absolute quantification of eicosanoids. Enzyme immunoassays (ELISA) or radioimmunoassays (RIA) do not deliver reliable results based on cross-reactivity, and gas chromatography/mass spectrometry (GC-MS) requires elaborate sample preparation, including derivatization, which increases the loss of analyte and consequently limits appropriate quantification [11]. To date, liquid chromatography/tandem mass spectrometry (LC-MS/MS) consuming organic solvents is the state of the art for analyzing and quantifying eicosanoids [12]. Supercritical fluid chromatography (SFC), already developed 60 years ago [13], uses supercritical CO_2_ as a mobile phase with favorable properties such as low viscosity and high diffusivity. The unique gas- and liquid-like abilities, which occur at a temperature and pressure above the thermodynamic critical point, allow higher flow rates even for small particle stationary phases with accompanying reduction of run times [14]. Supercritical CO_2_ is non-polar, but by the addition of varying amounts of polar modifiers such as methanol (MeOH) or isopropanol (IPA), separation of both polar and non-polar analytes is facilitated, which suggests SFC for analyzing oxidized polyunsaturated fatty acids ranging in polarity and occurring as stereoisomers. However, despite the development of robust instrumentations, so far only a few reports have been found that utilize SFC for oxylipin detection and quantification [15,16,17,18]. Here, we established and validated an SFC method coupled to a single quadrupole mass spectrometer to identify and quantify relevant eicosanoids. The applicability was then proven by the detection and quantification of PGs, TxB_2_, and LOX products in monocytes, platelets, and neutrophils, respectively.

## 2. Materials and Methods

### 2.1. Materials

Prostaglandin E_2_ (PGE_2_), prostaglandin D_2_ (PGD_2_), leukotriene B_4_ (LTB_4_), 6-*trans*-leukotriene B_4_ (t-LTB_4_), 6-*trans*-12-epi-leukotriene B_4_ (*trans*-epi-LTB_4_), 12-epi-leukotriene B_4_ (epi-LTB_4_), 20-hydroxyleukotriene B_4_ (20-OH-LTB_4_), thromboxane B_2_ (TxB_2_), 15(*S*)-hydroxyeicosatetraenoic acid (15(*S*)-HETE), 12(*S*)-hydroxyeicosatetraenoic acid (12(*S*)-HETE), 5(*S*)-hydroxyeicosatetraenoic acid (5(*S*)-HETE), prostaglandin B_1_ (PGB_1_), prostaglandin F_2α_ (PGF_2α_), and zileuton were purchased from Cayman Chemicals (Ann Abor, USA). MS-grade methanol (MeOH), isopropanol (IPA), and acetonitrile (ACN) were from VWR (Darmstadt, Germany), ammonium hydroxide from Honeywell (Charlotte, USA), and formic acid, diclofenac, and acetylsalicylic acid (ASS) from Merck KGaA (Darmstadt, Germany). Lipopolysaccharide (LPS), Histopaque-1077^®^, n-hexane, and methyl formate were from Merck (Darmstadt, Germany). L-glutamine and CaCl_2_ were purchased from Carl Roth (Karlsruhe, Germany).

### 2.2. SFC-MS Method Development

Supercritical fluid chromatography was carried out on an SFC/UHPLC-switching system (Shimadzu Corporation, Kyoto, Japan) equipped with one LC-30ADSF pump for liquid CO_2_, two LC-20ADXR pumps with DGU-20A5R degassing unit for modifier and makeup delivery, two SFC-30A back pressure regulators, a SIL-30AC autosampler, a CTO-20AC column oven, and an SPD-M20A as photodiode array. The system was coupled to an LCMS-2020 single quadrupole mass spectrometer with an electrospray ionization (ESI) source and the mass spectrometer was operated in a selected-ion monitoring (SIM) mode to detect the eicosanoids.

For initial column screening, 15 different columns with 12 different surface modifications were selected and their suitability was tested using a mixture of 11 common eicosanoids. The tested columns were Lux i-Cellulose 5 (3 × 100 mm, 3 µm), Lux i-Amylose 3 (2 × 150 mm, 3 µm), Gemini C6-Phenyl (4.6 × 250 mm, 5 µm), Kinetex HILIC (4.6 × 100 mm, 2.6 µm), Luna HILIC (4.6 × 250 mm, 5 µm), Luna NH_2_ (4.6 × 150 mm, 5 µm), Synergi Fusion-RP (4.6 × 250 mm, 4 µm), Synergi Hydro-RP (4.6 × 250 mm, 4 µm and 2 × 150 mm, 4 µm), Synergi Polar-RP (4.6 × 250 mm, 4 µm), Luna C5 (4.6 × 250 mm, 5 µm), Luna C8 (4.6 × 150 mm, 5 µm) from Phenomenex, Acquity BEH C18 (2.1 × 100 mm, 1.7 µm) and Torus Diol (2.1 × 100 mm, 1.7 µm) from Waters, and 60-5-Diol (4.6 × 250 mm, 5 µm) from Kromasil.

MeOH, IPA, or ACN supplemented with 0.1% (*v*/*v*) formic acid was used as mobile phase B (mobile phase A was sCO_2_). To cover the large range of polarity, the gradient started with 1% B for the first minute, rising to 5% B within 1 min and keeping it for 1 min, then increasing to 10% B within 1 min and keeping it for 1 min, then increasing modifier to 20% B within 2 min, keeping it for 1 min and increasing for the last time up to 30% B within 2 min and keeping it for 1 min followed by a 2 min re-equilibration window. The column temperature was set to 40 °C, the back pressure was 120 bar, and the flow rate was set depending on column dimensions ranging from 1.2 up to 3.5 mL/min. MeOH was used as a makeup solvent with a consistent flow rate of 0.2 mL/min.

The ESI source was operated in negative-ionization mode with a capillary voltage of 4.5 kV, source temperature of 350 °C, heat block temperature of 200 °C, desolvation line temperature of 250 °C, drying gas 8 L/min and nebulizing gas 1.5 L/min.

To achieve sufficient interference-free baseline separation of isobaric oxylipins, additional parameters were optimized upon the choice of the column. The final conditions were as followed: modifier IPA/ACN (7/3) + 0.1% formic acid (*v*/*v*), column temperature was set to 35 °C, back pressure was 100 bar, and the flow rate was set to 1.2 mL/min. The gradient was modified to: 0–1 min 5% B, 1–2 min 5 to 10% B, 2–4 min 10% B, 4–5 min 10 to 15% B, 5–6 min 15% B, 6–7 min 15 to 20% B, 7–9 min 20% B, 9–10 min 20 to 35% B, 10–12.5 min 35% B, 12.5–14 min 35 to 5% B, 14–15 min 5% B. 

For makeup and ESI optimization, a standard mixture of 11 eicosanoids (c = 50 ng/mL) was analyzed on three consecutive days, and MeOH, IPA, ACN, MeOH + 0.1% (*v*/*v*) NH_3_, and IPA + 0.1% NH_3_ were tested. ACN + 0.1% (*v*/*v*) NH_3_ lead to increased pump pressure and was therefore not evaluated. The makeup flow was set to 0.2 mL/min and the injection volume was 5 µL. 

Final ESI conditions consist of: capillary voltage 4.5 kV, source temperature 350 °C, heat block temperature 300 °C, desolvation line temperature 300 °C, drying gas 5 L/min, nebulizing gas 1.5 L/min, and makeup flow 0.05 mL/min. 

### 2.3. Method Validation

Linearity was examined using calibration curves ranging from 39 to 5000 ng/mL. Different regressions were evaluated by plotting the AUC against calibrator concentrations with a weighting factor of 1/x or 1/x^2^ and a linear or quadratic fit. To generate quality control (QC) samples, MeOH was spiked with all 11 authentic standards at four concentrations (lower limit of quantification QC (LLQC) = 78 ng/mL, low QC (LQC) = 250 ng/mL, medium QC (MQC) = 750 ng/mL, high QC (HQC) = 2000 ng/mL). Analysis was done on three consecutive days. Linearity was defined as the mean back-calculated concentration of ±15% of the nominal value, except for the LLOQ, where it should be within ±20%. Calculated QC samples should be within 20% of the nominal value. The calibration curve and QC samples were prepared by spiking MeOH with an analyte at a known concentration. Calibration curves ranging from 78 to 2500 ng/mL with linear regression and a weighting factor of 1/x provided the best and most robust results and were used in further experiments.

The LLOQ was defined as the lowest concentration level, which could be determined with acceptable accuracy and precision, and was corroborated by QC samples.

Accuracy and precision were calculated at four different concentration levels (LLOQ, three times LLOQ, 50% of calibration range, and 80% of upper calibration range) as required by EMA guidelines. Accuracy is expressed as a percentage of the nominal value. Acceptable ranges were defined as a maximum deviation of 15% from the nominal value except for LLOQ, where it should be within 20%. Precision is described as the closeness of repeated measurements and is expressed as relative standard deviation (%). Intraday accuracy and precision were determined by injection of six replicates of each QC sample. Interday values are the mean values of accuracy and precision determinations of three consecutive days. For recovery experiments, MeOH-inactivated neutrophils of three different donors were taken and spiked with all 11 authentic standards at two different concentrations followed by solid phase extraction, solvent evaporation, and reconstitution. In inactivated neutrophils without added standards, no endogenous levels were detected, and therefore inactivated neutrophils were considered a suitable matrix. Recovery is expressed as a percentage of nominal value. 

### 2.4. Purification of Primary Human Blood Cells

Monocytes, platelets, and neutrophils were isolated from citrated buffy coats obtained from the institute of transfusion medicine at the university hospital Greifswald from peripheral standard whole blood donations of healthy human adult male and female volunteers (Reg Nr BB014/14) as described [19]. The research was conducted following the Declaration of Helsinki. In brief, erythrocytes were removed by dextran sedimentation followed by a density gradient centrifugation on lymphocyte separation medium (Histopaque-1077^®^) gaining various cell types. Polymorphonuclear leukocytes, including neutrophils, were purged from remaining erythrocytes by hypotonic lysis, washed twice with phosphate buffered saline (PBS), and resuspended in PBS containing 0.1% (*w*/*v*) glucose (PG). 

Peripheral blood mononuclear cells (PBMCs) were washed twice with PBS and seeded in RPMI 1640 with 10% fetal calf serum (FCS), 2 mM L-glutamine, 100 U/mL penicillin and 100 µg/mL streptomycin for 90 min at 37 °C and 5% CO_2_. Adherent monocytes were washed twice with PBS, scraped with PBS, centrifuged, and resuspended in a culture medium.

Platelet-rich plasma was diluted with PBS pH 5.9 and washed with a mixture of equal parts saline and PBS pH 5.9. Platelets were resuspended in 5 mL PG buffer and counted using a Thoma chamber.

### 2.5. Cell Assays

Neutrophils (5 × 10^6^ cells/mL) were diluted in PG buffer containing 1 mM CaCl_2_ (PGC buffer) and incubated with inhibitor or 0.1% (*v*/*v*) vehicle for 10 min at 37 °C followed by stimulation with 2.5 µm A23187 with and without AA as described for respective experiments. After 10 min, product formation was stopped with 1 mL ice-cold MeOH, and as an internal standard 100 ng PGB_1_ was added. Samples were stored at −20 °C for at least 60 min for protein precipitation followed by centrifugation at 2000 rpm for 10 min at 4 °C. Supernatants were diluted with 8 mL acidified MilliQ pH 3.4 before purification via solid phase extraction (SPE). SPE cartridges (Chromabond C18ec, Machery Nagel) were equilibrated with 2 mL MeOH and 2 mL bi-distilled water. Samples were loaded and washed with 4 mL bi-distilled water and n-hexane and oxylipins were eluted with 2 mL methyl formate. The solvent was evaporated with a concentrator (Eppendorf, Hamburg) at 30 °C for 120 min, and resulting lipid mediators were resuspended in 50 µL MeOH.

Platelets (1 × 10^8^ cells/mL) were diluted in PGC buffer and incubated with indicated inhibitors or 0.1% vehicle for 5 min at room temperature followed by 60 s at 37 °C. Platelets were stimulated with 2.5 µm A23187 (±AA as indicated) for 5 min at 37 °C and stopped with 1 mL MeOH; 100 ng PGB_1_ was added and samples were processed as described above. In comparison to neutrophils and monocytes, platelet samples needed to be diluted 1:10 in MeOH before SFC injection.

Monocytes (1 × 10^6^/well) were seeded in 12-well plates in RPMI 1640 containing 10% FCS, 2 mM L-glutamine, 100 U/mL penicillin, and 100 µg/mL streptomycin and incubated at 37 °C, 5% CO_2_ with indicated inhibitor or vehicle (0.1% *v*/*v*) for 30 min. Cells were stimulated with LPS at indicated concentrations or with PBS as a vehicle for 24 h at 37 °C, 5% CO_2_. The supernatant was transferred into glass vials and 1 mL MeOH and 100 ng PGB_1_ were added. Samples were processed as described above. In all cell assays, absolute amounts of eicosanoids were calculated using the calibration curves, and values were normalized against the internal standard PGB_1_.

## 3. Results

### 3.1. Column Screening

**Figure 1:**
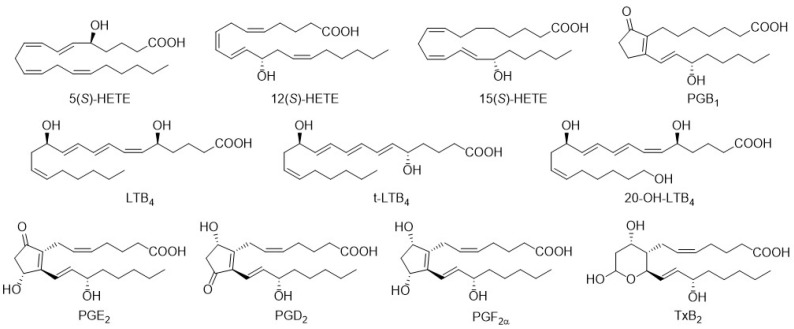
Included eicosanoids in the developed method.

To the best of our knowledge, there has been no published fully validated SFC-MS or SFC-MS/MS method that allows the separation of multiple mono-, di-, and trihydroxylated eicosanoids obtained from biological samples. Here, we aimed to develop an SFC-MS method that enables the separation and subsequent quantification of physiologically relevant eicosanoids (Figure 1). To select the appropriate stationary phase that provides sufficient separation of isobaric eicosanoids, 15 different columns were screened including columns with chiral selectors, HILIC columns, RP-columns with varying carbon chain lengths or polar modifications and columns with a diol or amino selectors. Note, the resolution of isobaric analytes was a critical parameter as it is required for detection by a single quadrupole mass spectrometer. 

**Figure 2:**
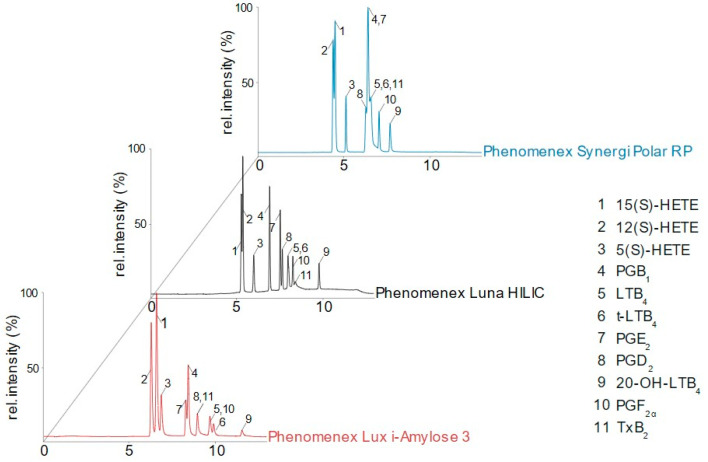
Chromatograms of selected columns. Exemplary, total ion count (TIC) of all measured *m*/*z* from a chiral column (LUX i-Amylose 3), an HILIC column (Luna HILIC), and a modified RP column (Synergi Polar RP) is shown.

On all tested columns, monohydroxylated metabolites of AA (5(*S*)-, 12(*S*)-, and 15(*S*)-HETE) eluted first, while the polar 20-OH-LTB_4_ showed the highest retention time. Hydrophobically modified silica columns (C5, C8, C18) provided low retention leading to peak broadening and coelution even at very low modifier concentrations, which led to exclusion from further testing. Acceptable, although not complete, separation of the analytes was obtained on Luna HILIC, Lux i-Amylose 3, and Synergi Polar-RP columns (Figure 2). Luna HILIC is a silica-based column with crosslinked diol groups, and the Synergi Polar-RP column is characterized by ether-linked phenyl residues with polar endcapping groups. On both columns, hydrophilic interactions with polar analytes are increased, which promotes their retention and enhances their analysis. However, while all three HETEs, PGE_2_, and PGD_2_ showed potential to reach sufficient separation by further method development, isomers of LTB_4_ coeluted and remained unresolved, which disqualified these columns to analyze 5-LOX products. Only stationary phases with chiral selectors such as the Lux i-Amylose 3 column were able to resolve LTB_4_ from its trans-isomer. Furthermore, isobaric PGE_2_ and PGD_2_, which coeluted from most of the tested columns, could be separated with sufficient resolution. Interestingly, on chiral columns, a reversed elution order of 15(*S*)- and 12(*S*)-HETE was observed (Figure 2). Based on the preliminary screening and advantages of the chiral selectors, method development was continued on the Lux i-Amylose 3 column. 

### 3.2. Method Development

**Figure 3:**
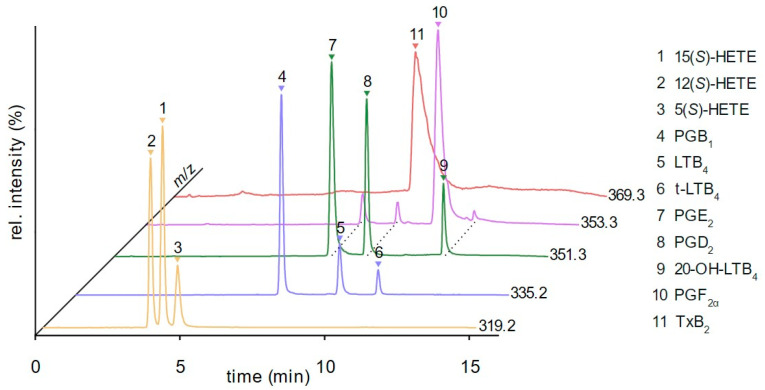
Chromatogram of 11 relevant eicosanoids after method development. Baseline separation of isobaric analytes shown by selected-ion monitoring (SIM) of *m*/*z* = 319.2 (5-HETE, 12-HETE, 15-HETE), *m*/*z* = 335.2 (PGB_1_, LTB_4_, t-LTB_4_), *m*/*z* = 351.3 (PGE_2_, PGD_2_, 20-OH-LTB_4_), *m*/*z* = 353.3 (PGF_2α_), and *m*/*z* = 369.3 (TxB_2_), which allows interference-free quantification. Conditions used in the final method: IPA/ACN (7/3) + 0.1% formic acid as modifier, oven temperature = 35 °C, BPR = 100 bar, u = 1.2 mL/min, Lux i-Amylose 3 column.

To achieve baseline separation of isobaric eicosanoids, modifier composition and column temperature were adjusted. While LTB_4_ and t-LTB_4_ had similar retention with IPA as a modifier, a binary modifier consisting of IPA/ACN (7/3) + 0.1% formic acid led to increased resolution due to higher retention of t-LTB_4_. In addition to the change to the binary modifier composition, the modifier gradient was adjusted to reach an optimal resolution. A decrease in column temperature from 40 to 35 °C further enhanced retention of t-LTB_4_ and improved separation of LTB_4_ isomers and concomitantly enhanced baseline separation of 12(*S*)- and 15(*S*)-HETE. Additionally, the resolution of PGE_2_ and PGD_2_ was further improved. Separation of all isobaric eicosanoids was achieved within 12 min (Figure 3). 

Note, PGE_2_, PGD_2_, and 20-OH-LTB_4_ undergo a non-enzymatic reductive degradation process as all three eicosanoids led to the detection of analytes in the SIM chromatogram of *m*/*z* = 353.3 but with a reasonable resolution to PGF_2α_. Capacity factors before and after the adaption of the chromatographic system are shown in Table 1. 

**Figure 4:**
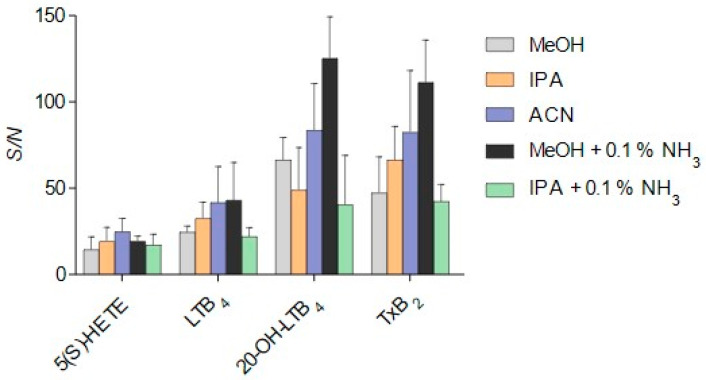
Impact of different makeup compositions on *S/N* after injection of a standard mixture with 50 ng/mL of each analyte. Measurements were taken on three consecutive days. Results are expressed as mean + SD.

SFC provides excellent compatibility with ESI due to the quick and complete evaporation of sCO_2_. However, by concentrating the analyte and especially at low modifier concentration, the risk of sample precipitation in the capillary is increased. Post-column addition of makeup solvents prevents this occurrence, enhances analyte ionization, and thus influences the corresponding *S/N* ratio. Therefore, the makeup solvent needs to be optimized for the analyte of interest. MeOH, IPA, and ACN with or without the addition of 0.1% NH_3_ were investigated (Figure 4). The tested solvents had no impact on the *S/N* ratio of monohydroxylated analytes, but the detection of more polar analytes was heavily affected by different solvents. IPA increased the *S/N* of LTB_4_ and TxB_2_ while decreasing 20-OH-LTB_4_. ACN improved the *S/N* of all tested analytes compared to MeOH but also increased variability. While the addition of ammonia to IPA led to inverse effects and reduced *S/N*, measurements with ACN + 0.1% NH_3_ as makeup could not be carried out due to a rapid increase in back and pump pressure. Satisfactory results were obtained using MeOH plus 0.1% NH_3_ (Figure 4) and therefore used as makeup for further method development. A makeup flow of 0.05 mL/min was sufficient to prevent analyte precipitation in the ESI unit while enhancing the *S/N*.

### 3.3. Method Validation

Oxylipin abundance differs in biological samples in species and concentration depending on the activity of metabolizing enzymes, substrate availability, and interindividual variations. Therefore, calibration curves need to cover a broad range of analyte concentrations. In the tested system, acceptable linearity was achieved in the range of 78–2500 ng/mL for all analytes. To assure accuracy and precision, separately spiked QC samples were measured against calibration curves on 3 consecutive days. According to EMA guidelines, QC samples were diluted to four concentration levels covering LLOQ (LLQC), 3xLLOQ (LQC), 30% (MQC), and 80% (HQC) of the calibration line. The method generates precise and accurate results (Table 2). Precision is expressed as the relative standard deviation of accuracy in a percentage amount of nominal value. Precision was in the range required by EMA except for intraday precision of t-LTB_4_ and 20-OH-LTB_4_ as it was slightly above the demanded 15% RSD. Accuracy was in the range of 85–115% of nominal value, except for 5(*S*)-HETE, where the accuracy of the LLQC sample was 123% (Table 2).

To assess the recovery rate of all analytes, including PGB_1_ as the internal standard, blank matrix samples (5 × 10^6^ neutrophils/mL) of three different donors were spiked with the standard at two different concentration levels. Proteins were precipitated by MeOH and samples were prepared as described in the methods. Recovery is expressed as a percentage amount of the nominal value detected after sample processing (Table 3). All analytes show reproducible results with low variance at both spiked concentrations. A noticeable trend is the higher recovery rate of cyclopentyl-containing metabolites. Especially PGB_1_ and PGE_2_ show values around 80%, while more linear structured molecules such as HETEs and LTB_4_ have a recovery in the range of 62–70%.

### 3.4. Method Application

**Figure 5:**
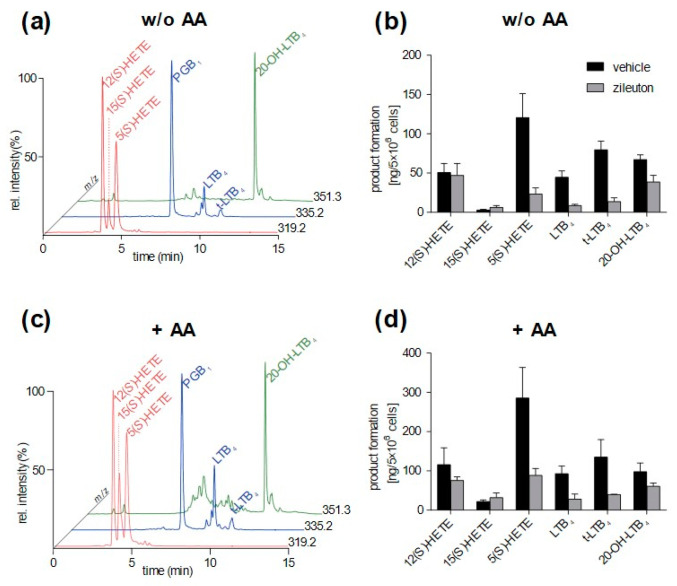
Quantitative analysis of selected oxylipins formed in neutrophils. Cells were preincubated (10 min) by zileuton (3 µm) or vehicle (0.1% *v*/*v*) followed by 10 min stimulation with 2.5 µm A23187 without AA (**a**,**b**) or with 5 µm AA (**c**,**d**). Representative SIM chromatograms are shown in (**a**,**c**) and (**b**,**d**) presenting quantitative data of analyzed oxylipins formed via 5-, 12-, and 15-LOX pathways. Values are expressed as mean + SEM (*n* = 3).

The applicability of the validated method was tested on eicosanoids formed in different types of primary human blood cells. Neutrophils convert AA to 5(*S*)-HETE and LTB_4_ via the 5-LOX pathway [3]. While t-LTB_4_ and t-epi-LTB_4_ are formed via nonenzymatic processes from the unstable epoxide LTA_4_, LTB_4_ biosynthesis requires LTA_4_H activity that hydrolyzes LTA_4_ to LTB_4_ [20]. Especially in neutrophils, LTB_4_ is rapidly oxidized at the omega-end by CYP enzymes to 20-OH-LTB_4_ [21]. Therefore, neutrophils were used to test the method’s applicability for 5-LOX products. Due to impurities of platelets and eosinophils within the neutrophil population, we were able to detect 12(*S*)-HETE and 15(*S*)-HETE, which were generated via 12-LOX (platelets) and 15-LOX (eosinophils), respectively (Figure 5). Without the addition of exogenous AA, all analytes could be quantified within the validated range, and zileuton, a specific 5-LOX inhibitor reduced 5-LOX products, which confirms enzymatic eicosanoid biosynthesis (Figure 5a,b). As expected, the formation of 12(*S*)-HETE and 15(*S*)-HETE remained unaffected by zileuton. The addition of exogenous AA enhanced the detection of 15(*S*)-HETE, but at the same time, impurities, especially in the SIM chromatogram of *m*/*z* = 335.2 and *m*/*z* = 351.3 and are potentially evoked by autoxidative products of AA, hamper analysis of LTB_4_ isomers (Figure 5c). Furthermore, a reasonable quantification of 5(*S*)-HETE cannot be guaranteed as the detected amounts exceeded the validated range, but even here zileuton potently reduces 5-LOX product formation (Figure 5d).

**Figure 6:**
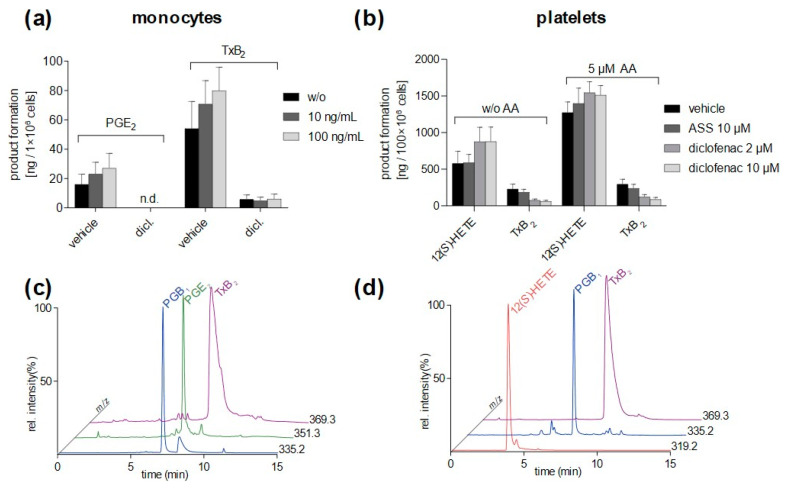
(**a**) TxB_2_ and PGE_2_ formation in monocytes generated by LPS stimulation (10 and 100 ng/mL). Eicosanoid biosynthesis was inhibited by pretreatment with diclofenac (2 µm). (**b**) Platelets were resuspended in PGC buffer, preincubated by COX inhibitors ASS (10 µm) or diclofenac (2 and 10 µm), and 12(*S*)-HETE and TxB_2_ formation in platelets was initiated by 2.5 µm A23187 ± 5 µm AA. Samples were diluted at 1:10 to stay within the validated range. While COX-derived TxB_2_ formation was inhibited, 12(*S*)-HETE was formed by 12-LOX independent of COX inhibitors. Values are expressed as mean + SEM (*n* = 3). (**c**,**d**) Representative SIM chromatograms are shown for PGE_2_ and TxB_2_ formation in monocytes (**c**) and 12(*S*)-HETE and TxB2 in platelets (**d**). PGB_1_ was added exogenously as an internal standard.

To evaluate the method applicability for eicosanoids biosynthesized by COXs, monocytes were stimulated by LPS at different concentrations for 24 h, and diclofenac was used as a COX inhibitor to demonstrate enzymatic formation. PGE_2_ and TxB_2_, both downstream metabolites of the COX pathway were formed in LPS-stimulated monocytes and diclofenac abolished or reduced the biosynthesis, respectively (Figure 6a). Note, monocytes constitutively express COX-1 and lead to PGE_2_ and TxB_2_ formation also in non-stimulated cells. COX product formation was elevated by LPS stimulation, potentially by elevated COX-2 expression. In all assay setups, PGE_2_ and TxB_2_ could be quantified in the validated range.

Platelets are known to biosynthesize high levels of 12(*S*)-HETE and TxB_2_ due to the strong activity of 12-LOX and COX/TXs, respectively. Note, to keep concentration levels within the validated range, samples needed to be diluted 1:10 before measurements, and data presented in Figure 6b show the final concentration in the biological sample. Acetylsalicylic acid (ASS), as well as diclofenac significantly reduced TxB_2_ formation, while 12-LOX generated 12(*S*)-HETE remained unaffected. In experiments conducted without exogenous AA, diclofenac treatment slightly elevated 12(*S*)-HETE formation, potentially due to a substrate shift (Figure 6b).

## 4. Discussion

Eicosanoids are potent signaling molecules, biosynthesized in various tissues and cells [22]. However, identification and quantification remain problematic due to similarities in structure and related physical and chemical properties, the occurrence of autoxidation during sample preparation, and low abundance in biological matrices. To enhance the understanding of eicosanoid concentration in various tissues and associated disease progression, reliable, specific, and sensitive analytical methods are essential and of great interest. During the last decades, reliable UHPLC-MS platforms were developed to analyze a variety of oxylipin species, including eicosanoids [12,23]. With the development of robust instrumentation and coupling to MS, also SFC became a powerful tool to solve difficult separation problems [24]. Therefore, changing the mobile phase to supercritical fluids might be advantageous over chromatographic separation by organic solvents. For example, stereoisomers were commonly separated by chiral or normal phases. While chiral columns generally require long equilibration times, non-polar eluents have the drawback of problematic ionization in the mass spectrometer [25]. Supercritical CO_2_ shares comparable polarity with hexane but is miscible with polar solvents, which allows the separation of analytes covering a broad polarity range with exceptional MS compatibility. Furthermore, high diffusivity and low viscosity of supercritical CO_2_ enable higher flow rates with sub 2 µm particle columns, which reduces run time and associated low consumption of organic solvents compared to HPLC analysis [14]. So far, very few reports present analytical platforms that use SFC-MS for oxylipin analysis. Kumari et al. [15] published first a method including four PGs and LTB_4_, which facilitates very short run times (< 3 min), but requires a tandem MS for analyses of isobaric analytes. Furthermore, despite the detection of few eicosanoids, the reported method did not provide sufficient separation of TxB_2_ and had to be removed from the analysis. An improved method was published by Berkecz, et al. [16] that enables the separation of 20 isobaric oxylipins within 8 min. They compared their SFC method with an established RP-UHPLC method and concluded that due to better separation and higher sensitivity, the robust RP-UHPLC platform is still the preferred method. The Wheelock lab developed an SFC-MS/MS platform to ensure quantitative metabolic profiling of oxylipins that derive from C18-fatty acids. The use of chiral stationary phases coupled to tandem-MS allowed the separation of 103 octadecanoids and demonstrates the advantages of using supercritical CO_2_ as a mobile phase in oxylipin analysis [18].

The overall aim of this study was to show the feasibility of SFC to separate eicosanoids covering a wide range of polarity and to demonstrate applicability in daily laboratory practice by coupling the SFC to a single quadrupole mass detector. The presented method allows analysis of 11 relevant eicosanoids covering mono-, di-, and trihydroxylated derivates in less than 13 min without the need for multiple reaction monitoring due to isobaric baseline separation. Among all tested columns, only stationary phases with chiral selectors were suitable, while modified normal and polar derivatized RP-phases showed coelution of LTB_4_ isomers. On HILIC and polar modified RP columns, predominant hydrophilic interactions occur between the carboxy and hydroxy groups of eicosanoids and the hydroxy groups of the stationary phase. The sugar-polymer-based chiral column (Lux i-Amylose 3; amylose tris(3-chloro-5-methylphenylcarbamate) adds additional hydrophobic retention, steric hindrance, and polar hydrogen–π interactions and therefore enhances retention and isomer separation. Moreover, regioisomers as 5(S)-, 12(S)-, and 15(S)-HETE are only separated sufficiently on the Lux i-Amylose 3 column. The combination of polar and aromatic selectors on polysaccharides is required to reach the sample resolution of stereo- and regioisomers. Similar results were reported before where 1-aminoanthracene and 2-picolylamine substituted columns proved to be superior over other polar modified phases without aromatic structures [15,16]. Among chiral polysaccharide selectors, amylose-based columns were advantageous over cellulose for oxylipin isomer separation. This is an observation that was described before [18,26,27,28,29]. According to EMA guidelines, the method was validated towards linearity, LLOQ, accuracy, precision, and recovery. The range was set from 78 to 2500 ng/mL to ensure reliable quantification of analytes. Due to the complexity of biological matrices and the detection limitation of a single quadrupole MS, LLOQ was set higher than an *S*/*N* of 10 to exclude false positive detection. Transmission on a system containing a triple quadrupole that allows tandem MS potentially lowers the LLOQ due to multiple reaction monitoring and reducing noise levels. The recovery rate varies between 58.9 and 81.6% with PGB_1_ having the overall highest rate while linear structured eicosanoids such as HETEs and LTB_4_ isomers have a recovery of <70%. Remarkably, all analytes containing cyclopentyl moiety showed recovery rates >70%. This gap could be the result of the residual activity of human neutrophils, which served as a biological matrix in our experiments and are strongly enriched in buffy coats used for blood cell isolation. Despite inactivation with MeOH, insufficient protein denaturation could occur leading to metabolism or degradation of spiked oxylipins. Other explanations might be the different affinity of the eicosanoids to SPE material or insufficient elution by methyl formate that may reduce recovery. Nevertheless, due to similar elution properties to LTB_4_ isomers, PGB_1_ is often used as an internal standard in eicosanoid analysis and is also successfully deployed to quantify monohydroxylated products [30].

Many eicosanoids are formed in blood cells as neutrophils, monocytes, macrophages, or platelets. Therefore, the applicability of the presented and validated method was confirmed by *in vitro* cell assays. LTs are predominately biosynthesized in neutrophils and act as inflammatory mediators of the innate immune response. With the present SFC-MS method, 5-LOX products (5(*S*)-HETE, LTB_4_, t-LTB_4_, 20-OH-LTB_4_) could be quantified in the range of 78–2500 ng/mL. While the nonenzymatic formed 6-*trans*-isomers of LTB_4_ coeluted behind LTB_4_, an additional peak was detected shortly before LTB_4_, although without affecting the quantification of LTB_4_. Testing authentic standards suggests elution of 12-*epi* LTB_4,_ which is often coeluted with LTB_4_ and is overlooked in many applied RP-HPLC methods. The detected amounts of 5-LOX metabolites in A23187-challenged neutrophils were comparable with values obtained by UHPLC-MS/MS (e.g., LTB_4_, ~30 ng/5 × 10^6^ cells) [31]. The applicability for inhibitor studies was confirmed by zileuton, which reduced 5-LOX product formation of endogenously metabolized AA as well as products that were formed from exogenously added AA. Furthermore, an inhibitor-mediated decrease in metabolite biosynthesis confirms enzyme involvement and excludes autoxidation. Note, 12(*S*)- and 15(*S*)-HETE are mainly biosynthesized by 12- and 15-LOX impurities from platelets and eosinophils, respectively. As expected, the biosynthesis remained unaffected by zileuton. COX-derived products can be analyzed in monocytes and platelets as both cells express COX-1. In monocytes, COX-2 expression can be elevated by LPS stimulation [32], which was also confirmed in our experiments by the detection of elevated levels of PGE_2_ and TxB_2_. The amounts of PGE_2_ quantified by the SFC-MS method (15–30 ng/1 × 10^6^ cells) were comparable to the levels analyzed by LC-MS/MS in LPS-stimulated monocytes [33]. COX-derived biosynthesis was demonstrated by diclofenac, a potent unselective COX-1/2 inhibitor. Compared to monocytes, platelets form huge amounts of TxB_2_ and 12(*S*)-HETE, that exceed the validated range of our method, especially when cells were stimulated by exogenous AA. Therefore, samples had to be diluted (1:10) before analysis by the presented method to stay within the range and thus generate reliable data. Enzymatic formation of TxB_2_ was demonstrated by reversible and irreversible COX inhibitors, diclofenac and ASS, respectively. In all presented cell assays, we were able to demonstrate the applicability of the novel validated SFC-MS method, which can be routinely used for inhibitor studies or to investigate eicosanoid biosynthesis during inflammatory processes.

In conclusion, a novel SFC-MS-based method was established and validated to successfully quantify 11 eicosanoids, with a relevant impact on physiological and pathophysiological conditions. Chiral columns outperformed RP, NP, or HILIC columns, and modifier adaption enables baseline separation of isobaric species, which allows eicosanoid detection by a single quadrupole MS. Soon, chiral SFC coupled to tandem-MS will improve the opportunity to quantify simultaneously more than 100 oxylipin species and thus offers a platform to expand our knowledge of the oxylipin metabolome in health and disease.

## Figures and Tables

**Table 1:** Capacity factors of eicosanoids included in the method and their corresponding *m/z* values for detection by mass spectrometry.

Compound	*m*/*z*	*k* Screening Condition	*k* Final Method Condition
12(*S*)-HETE	319.2	9.7	5.3
15(*S*)-HETE	319.2	10.2	6.1
5(*S*)-HETE	319.2	10.7	6.8
PGB_1_	335.2	13.3	11.1
LTB_4_	335.2	15.5	14.5
t-LTB_4_	335.2	15.8	16.7
PGE_2_	351.3	13.1	11.7
PGD_2_	351.3	14.2	13.7
20-OH-LTB_4_	351.3	18.6	18.3
PGF_2α_	353.3	15.4	16.2
TxB_2_	369.3	14.3	13.3

**Table 2:** Accuracy and precision of method validation. Accuracy is expressed as the percentage amount of added analyte to the blank. Precision is reported as the coefficient of variation. QC samples are MeOH spiked with all 11 eicosanoids at 4 different concentrations. Concentration levels: LLQC = 78 ng/mL, LQC = 250 ng/mL, MQC = 750 ng/mL, HQC = 2000 ng/mL.

		Accuracy (%)		Precision (RSD%)	
Compound	QC	Intraday (*n* = 6)	Interday (*n* = 3)	Intraday (*n* = 6)	Interday (*n* = 3)
12(*S*)-HETE	LLQC	109.6	109.0	5.1	0.5
	LQC	91.0	98.0	6.8	6.8
	MQC	93.0	93.5	6.6	4.5
	HQC	89.3	93.7	7.3	4.8
15(*S*)-HETE	LLQC	110.7	112.4	5.7	2.6
	LQC	91.3	99.8	7.0	8.1
	MQC	94.4	95.6	7.3	3.0
	HQC	90.4	95.4	9.3	5.4
5(*S*)-HETE	LLQC	123.3	113.6	4.1	8.4
	LQC	87.8	96.6	6.4	8.9
	MQC	92.9	92.6	10.8	2.7
	HQC	89.5	94.9	12.6	7.0
PGB_1_	LLQC	94.1	100.0	8.8	7.3
	LQC	93.1	95.5	7.8	6.7
	MQC	95.0	93.3	4.4	4.0
	HQC	102.9	97.8	6.2	5.9
LTB_4_	LLQC	114.5	110.7	2.0	4.8
	LQC	97.0	100.7	4.9	3.4
	MQC	102.0	98.9	2.7	2.7
	HQC	103.6	101.1	4.5	3.4
t-LTB_4_	LLQC	107.4	98.6	5.9	9.2
	LQC	102.3	99.6	3.9	15.3
	MQC	101.9	98.9	5.3	5.4
	HQC	104.7	100.5	4.4	4.4
PGE_2_	LLQC	119.0	104.6	4.1	12.0
	LQC	94.7	92.4	4.4	3.9
	MQC	98.4	92.6	3.2	5.6
	HQC	97.9	95.7	9.4	2.6
PGD_2_	LLQC	111.9	104.4	5.5	6.2
	LQC	96.7	95.9	7.1	5.0
	MQC	103.1	96.1	8.5	9.9
	HQC	102.4	99.5	8.0	4.8
20-OH-LTB_4_	LLQC	110.2	106.9	6.7	16.6
	LQC	97.1	92.0	10.0	11.8
	MQC	95.8	88.6	9.0	12.1
	HQC	97.9	92.0	9.9	8.7
PGF_2α_	LLQC	112.7	107.7	7.2	5.0
	LQC	96.3	96.7	5.4	3.8
	MQC	114.0	102.3	10.9	11.1
	HQC	114.0	110.0	3.2	5.0
TxB_2_	LLQC	117.2	106.3	7.2	9.1
	LQC	99.4	97.8	5.5	2.7
	MQC	105.0	96.4	8.8	10.4
	HQC	113.2	103.8	5.4	9.7

**Table 3:** The recovery rate is expressed as a percentage amount of analyte at two concentration levels (I = 50 ng, II = 125 ng) found in spiked neutrophil samples of three donors. Results are shown as mean ± SD.

	**Recovery** (**%**)	
	**I**	**II**
12(*S*)-HETE	63.3 ± 5.2	64.2 ± 3.3
15(*S*)-HETE	65.3 ± 3.3	66.5 ± 3.0
5(*S*)-HETE	73.0 ± 7.6	69.8 ± 8.7
PGB_1_	80.4 ± 5.3	81.6 ± 2.3
LTB_4_	61.8 ± 3.5	63.6 ± 1.9
t-LTB_4_	64.4 ± 3.1	60.9 ± 0.7
PGE_2_	73.0 ± 3.4	80.4 ± 2.4
PGD_2_	75.8 ± 4.3	74.9 ± 1.5
20-OH-LTB_4_	60.2 ± 4.0	58.9 ± 0.7
PGF_2α_	69.8 ± 6.0	73.0 ± 5.8
TxB_2_	70.3 ± 3.6	76.6 ± 3.5

## Data Availability

Not applicable.
